# Peer Review of “Technologies to Support Assessment of Movement During Video Consultations: Exploratory Study”

**DOI:** 10.2196/32263

**Published:** 2021-09-24

**Authors:** Immanuel Victor George

**Affiliations:** 1 NHS WWL Foundation Trust Wigan United Kingdom

**Keywords:** tele-rehabilitation, video-consultations, assessment of movement, eHealth, technology, desktop robots, wide-angle webcams, physical health, rehabilitation, remote, assessment, assistive technology, evaluation, framework, webcam, telehealth, robots

*This is a peer-review report submitted for the paper “Technologies to Support Assessment of Movement During Video Consultations: Exploratory Study.”*

## Round 1 Review

### General Comments

This paper [1] is an exploratory study on technologies to support video consultations assessing movement. It is not clear whether it explored the technology itself or the process of using various technologies.

### Specific Comments

#### Major Comments

1. It is not clear why 4 specific devices were chosen and there is no explanation of the most widely used software. Is it technology or device exploration?

2. I was unable to identify clearly whether the hypothetical patients were physiotherapists or family members. Were the hypothetical patients briefed on what they should present for inference, or was the clinical condition identified as they presented?

3. How many hypothetical patients took part in the study?

#### Minor Comments

4. The experiences of the hypothetical patients were not detailed.
